# Reclassifications of ischemic stroke patterns due to variants of the Circle of Willis

**DOI:** 10.1177/17474930211048381

**Published:** 2021-10-05

**Authors:** Ida Rangus, Lennart S Milles, Ivana Galinovic, Kersten Villringer, Heinrich J Audebert, Jochen B Fiebach, Christian H Nolte, Hebun Erdur

**Affiliations:** 1 Department of Neurology, Charité – Universitätsmedizin Berlin, Corporate Member of Freie Universität Berlin and Humboldt Universität zu Berlin, Berlin, Germany; 2 Department of Neurology, Universitätsklinikum Essen, Essen, Germany; 3 Center for Stroke Research Berlin, Berlin, Germany; 4 Berlin Institute of Health (BIH), Berlin, Germany

**Keywords:** Circle of Willis, stroke patterns, neuroanatomy

## Abstract

**Background:**

Variants of the Circle of Willis (vCoW) may impede correct identification of ischemic lesion patterns and stroke etiology. We assessed reclassifications of ischemic lesion patterns due to vCoW.

**Methods:**

We analyzed vCoW in patients with acute ischemic stroke from the 1000+ study using time-of-flight magnetic resonance angiography (TOF MRA) of intracranial arteries. We assessed A1 segment agenesis or hypoplasia in the anterior circulation and fetal posterior cerebral artery in the posterior circulation. Stroke patterns were classified as one or more-than-one territory stroke pattern. We examined associations between vCoW and stroke patterns and the frequency of reclassifications of stroke patterns due to vCoW.

**Results:**

Of 1000 patients, 991 had evaluable magnetic resonance angiography. At least one vCoW was present in 37.1%. VCoW were more common in the posterior than in the anterior circulation (33.3% vs. 6.7%). Of 238 patients initially thought to have a more-than-one territory stroke pattern, 20 (8.4%) had to be reclassified to a one territory stroke pattern after considering vCoW. All these patients had fetal posterior cerebral artery and six (30%) additionally had carotid artery disease. Of 753 patients initially presumed to have a one-territory stroke pattern, four (0.5%) were reclassified as having more-than-one territory pattern.

**Conclusions:**

VCoW are present in about one in three stroke patients and more common in the posterior circulation. Reclassifications of stroke lesion patterns due to vCoW occurred predominantly in the posterior circulation with fetal posterior cerebral artery mimicking multiple territory stroke pattern. Considering vCoW in these cases may uncover symptomatic carotid disease.

## Introduction

Identifying stroke lesion patterns and affected territories supplied by neck arteries is an essential component in determining ischemic stroke etiology. More-than-one territory stroke patterns suggest a proximal embolic source, e.g. of cardiac or aortal origin, whereas single territory stroke patterns point to large or small vessel disease.^1^ Assigning stroke lesions to the corresponding arterial territories is usually carried out using pre-set brain maps due to practicality.^2^ However, since the Circle of Willis (CoW) connects all three major cerebral territories (left anterior, right anterior, and posterior), classifying stroke lesion patterns based solely on brain maps without considering variants of the Circle of Willis (vCoW) may lead to false assignment of affected arterial territories.^3^ Taking vCoW into account may lead to clinically relevant reclassifications of stroke patterns.

## Aims

Establishing the frequency of different vCoW could help to distinguish stroke locations that are more prone to misclassifications. Here, we analyzed the frequency of two vCoW in patients with ischemic stroke using time-of-flight magnetic resonance angiography (TOF MRA): (a) agenesis or hypoplastic A1 segment of the ACA in the anterior circulation (anterior vCoW) and (b) fetal posterior cerebral artery (fPCA) in the posterior circulation. Furthermore, we examined stroke lesion patterns (classified as one and more-than-one territory stroke pattern) in patients with complete Circle of Willis (cCoW) and vCoW. Finally, we examined the rate of reclassifications from more-than-one territory to one territory and vice versa, following reattributions of stroke lesions to the corresponding cerebral artery after identifying vCoW.

## Methods

### Study design and study population

MRI data were obtained from the prospective observational cohort study 1000+ (Clinical trials.gov identifier: NCT00715533), which was approved by the ethics committee of the Charité – Universitätsmedizin Berlin (EA4/026/08). The study enrolled consecutively admitted patients with transient ischemic attack or ischemic stroke to our stroke unit (Department of Neurology, Charité – Universitätsmedizin Berlin, Campus Benjamin Franklin, Berlin, Germany) from February 2011 to April 2013.^4^ The inclusion criteria were onset of acute ischemic stroke symptoms within the last 24 h of admission to the emergency department, meeting general MRI eligibility criteria and age ≥18 years.^4^ Additionally, only patients with a complete MRA were included into the current subanalysis.

Stroke severity was assessed using the National Institutes of Health Stroke Scale (NIHSS) with higher scores indicating greater severity (range: 0–42). Functional outcome at discharge was evaluated by modified Rankin Scale (mRS), a seven-point scale ranging from “0” (no neurological deficit) to “6” (death). We also recorded history of arterial hypertension, diabetes mellitus, hypercholesterinemia, coronary heart disease, atrial fibrillation, and smoking. Only patients with a diffusion weighted imaging (DWI) lesion that were previously included in the substudy by Erdur et al. (n = 1000) were analyzed in this substudy.^3^

### Neuroimaging and assessment of the variants of the Circle of Willis

All exams were acquired using 3-Tesla magnetic resonance imaging (MRI); Tim Trio 3T whole-body system (Siemens Healthcare, Erlangen, Germany) using a 12-channel receive radiofrequency (RF) coil (Siemens Healthcare). Extracranial neck arteries were either assessed by MRA, computed tomography angiography (CTA), or duplex ultrasound. The CoW was assessed using TOF MRA; voxel size 0.52 × 0.52 × 0.65 mm, matrix size 312 × 384 × 127 voxels, TR/TE = 22/3.86 ms, respectively, acquisition time 3:50 and flip angle 18°.

LSM mapped and characterized all lesions on DWI, FLAIR, and anatomical variations of cerebral vessels while blind to all clinical data except name and sex under continuous supervision by HE. All inconclusive imaging findings were discussed by HE, LSM, JBF and CHN for definitive allocation.

Stroke lesion patterns and vCoW were previously evaluated in a substudy by Erdur et al. using the data from the 1000+ study.^3^ The current work represents a subanalysis of this data set. We examined the frequency of two vCoW that occur most commonly.^5,6^ In the anterior circulation, we assessed the agenesis or hypoplasia of the A1 segment of the anterior cerebral artery (ACA) – anterior vCoW. This variant was identified when one A1 segment was significantly narrower than the contralateral A1 segment or absent. Consecutively, blood supply to the A2 segment ipsilateral to the underdeveloped A1 segment was manly provided from the contralateral A1 segment via anterior communicating artery (AComA). In the posterior circulation, we defined fPCA where posterior communicating artery (PComA) was significantly larger than the P1-segment of the ipsilateral posterior cerebral artery (PCA). Vice versa, the adult PCA was defined as P1-segment of the PCA being larger than the ipsilateral PComA. In cases with nearly the same diameters of the PComA und P1-segment, the posterior circulation was labeled as transitional, as previously described in the literature.^7^

We conducted separate comparisons of patients with vCoW and cCoW in the posterior and anterior circulation in terms of demographics, medical history, stroke severity (NIHSS on admission), and early functional outcome (mRS at discharge). Patients with only transitional posterior circulation were excluded from the comparison of vCoW due to the small sample size and because it was not possible to assign transitional PCA to either adult PCA or fetal PCA variant, owing to distinct hemodynamics of each variant. Patients with fPCA on one side and either adult or transitional PCA on the other side were considered as fPCA patients for this particular analysis.

Hyperintense lesions in the DWI and low apparent diffusion coefficient (ADC) were considered as acute ischemic lesions. Chronic ischemic lesions were assessed using fluid attenuated inversion recovery (FLAIR) sequence. Only acute ischemic lesions were considered for determining stroke patterns and affected arterial territories. Arterial territories were divided into left and right anterior (middle cerebral artery (MCA) + ACA territory of each side, originating from the internal carotid artery (ICA)) and posterior territory (PCA territories and vertebrobasilar system). Allocating stroke lesions to the corresponding arterial territories was initially performed using brain maps according to Tatu et al. with no regard to possible vCoW.^2,3^

**Figure 1.**
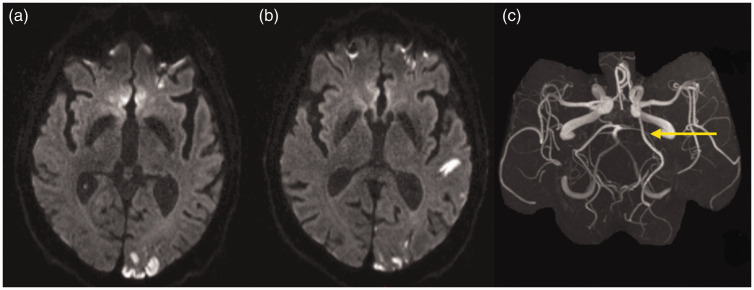
Reclassification from more-than-one to one territory stroke pattern. Example of reclassification from more-than-one to one territory stroke pattern in a patient with ischemic stroke affecting left PCA, ACA (a) and MCA territory (b) in the DWI sequence. Due to fPCA on the left side (indicated with an arrow in figure c), all stroke lesions are located within the arterial supply of the left ICA (anterior territory).

**Figure 2.**
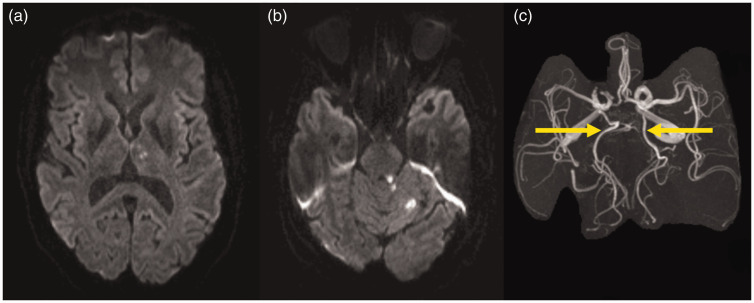
Reclassification from one territory to more-than-one territory stroke pattern. Example of reclassification from one territory to more-than-one territory stroke pattern in a patient with ischemic stroke in the left thalamus (a) and in the pons and cerebellum (b) in the DWI sequence. MRA revealed fPCA on both sides (indicated with arrows in figure c). Since thalamus arterial supply stems from the fPCA, thalamic lesion is located within the arterial supply of the ICA (anterior territory) while other lesions are located within the territory supplied by the vertebrobasilar system (posterior territory).

Stroke lesion patterns were classified as one and more-than-one territory stroke pattern. We examined the frequency of different stroke patterns with no regard to vCoW. After identifying vCoW, we examined the rate of reclassifications from more-than-one territory to one territory stroke pattern. For example, in a patient with ipsilateral temporal and occipital lesions, the lesions were labeled as more-than-one territory pattern. This had to be corrected to a one territory pattern after ipsilateral fPCA was identified (Figure 1). Reversely, reclassification from one to more-than-one territory pattern was conducted in cases where simultaneous PCA strokes and cerebellar strokes were seen and had been considered to belong to the same (posterior) territory. However, in patients with fPCA, the PCA stroke belonged to the anterior circulation, and consequently, a reclassification into more-than-one territory group was conducted (Figure 2). In addition, we examined how often ipsilateral fPCA is present in patients with isolated lesions in the PCA territory. In the presence of fPCA, isolated lesions in the PCA territory were re-allocated from the posterior to the (ipsilateral) anterior vascular territory.

### Statistics

SPSS Version 25.0. (IBM, Armonk, NY, USA) was used for statistical analyses. Metric data are presented as medians with interquartile range (IQR) due to non-normal distribution and were compared using Mann-Whitney U-Test. Categorical data were compared using chi-squared test or Fisher’s exact test as appropriate. All tests were performed at *α* = 0.05 level of confidence and were two sided.

## Results

### Characteristics of the study population

Of 1000 included patients, 991 had complete data sets of MRAs fully eligible for further analyses. MRA was not available in nine patients, either due to motion artifacts or because examination had to be stopped prematurely. Cases without MRA were excluded from further analyses. Of these 991 patients, 448 (45.2%) were female and median age was 74 years (IQR: 66–81). History of arterial hypertension was present in 86.2%, 29.7% had atrial fibrillation, 26.3% had diabetes mellitus type 2, and 18.8% had a history of coronary heart disease. Furthermore, 20.1% of patients reported being a smoker. Median NIHSS on admission was 4 (IQR: 2–8).

### Frequency of vCoW

At least one variant was present in 37.1% of patients. In the posterior circulation, 33.3% of patients had a fPCA. Unilateral fPCA was more common than bilateral fPCA (25.6% vs. 7.7%, p < 0.001) and fPCA was observed significantly more often on the right side than on the left side (14.9% vs. 10.7%, p < 0.001). Transitional variant was seen in 1.8% on the left side, in 1.3% on the right side, and in 0.2% on both sides. Women were significantly less likely to present with adult PCA than men. No further differences in terms of demographics and clinical characteristics were observed according to vCoW in the posterior circulation (Table 1). In the anterior circulation, 6.7% of the patients had vCoW, which was more common on the right side than on the left side (4.9% vs. 1.8%, p < 0.001). Patients with anterior vCoW and cCoW were similar according to demographic and clinical characteristics (Table 2).

**Table 1.** Comparison of the demographics and cardiovascular risk factors in patients with posterior vCoW (fPCA) and posterior cCoW (adult PCA). Patients with transitional posterior circulation (n=21) were excluded from this analysis.

	Posterior vCoW (fPCA) n = 330	Posterior cCoW (adult PCA) n = 640	p value^a^
Age (years); median (IQR)	74 (66–81)	74 (66–81)	0.833
Sex, no. female (%)	**166 (50.3)**	**270 (42.2)**	**0.017**
Arterial hypertension (%)	284 (86.1)	552 (86.3)	1.000
Diabetes mellitus type 2 (%)	74 (22.4)	178 (27.8)	0.076
Hypercholesterinemia (%)	178 (53.9)	361 (56.4)	0.495
Coronary heart disease (%)	52 (15.8)	130 (20.3)	0.099
Atrial fibrillation^b^ (%)	103 (31.2)	183 (28.6)	0.414
Smokers (%)	61 (18.5)	133 (20.8)	0.446
NIHSS on admission; median (IQR)	4 (2–8)	4 (2–8)	0.950
mRS at discharge; median (IQR)	2 (1–3)	2 (1–3)	0.179

NIHSS: National Institutes of Health Stroke Scale; mRS: modified Rankin Scale; PCA: posterior cerebral artery; fPCA: fetal posterior cerebral artery; cCoW: complete Circle of Willis; vCoW: Variants of the Circle of Willis.

a Mann-Whitney U-test for continuous variables and chi-squared test or Fishers exact test for categorical variables.

b Both history of atrial fibrillation and newly diagnosed atrial fibrillation on admission or during hospital stay.

**Table 2.** Comparison of the demographics and cardiovascular risk factors in patients with anterior vCoW and anterior cCoW.

	Anterior vCoW n = 67	Anterior cCoW n = 924	p value^a^
Age, (years); median (IQR)	75 (69–84)	74 (65–81)	0.056
Sex, no. female (%)	30 (44.8)	418 (45.2)	0.942
Arterial hypertension (%)	60 (89.6)	794 (85.9)	0.469
Diabetes mellitus type 2 (%)	19 (28.4)	242 (26.2)	0.774
Hypercholesterinemia (%)	35 (52.2)	517 (56.0)	0.611
Coronary heart disease (%)	10 (14.9)	176 (19.0)	0.427
Atrial fibrillation^b^ (%)	20 (29.9)	274 (29.7)	0.973
Smokers (%)	10 (14.9)	189 (20.5)	0.344
NIHSS on admission; median (IQR)	5 (2–8)	4 (2–8)	0.440
mRS at discharge; median (IQR)	2 (1–4)	2 (1–3)	0.218

NIHSS: National Institutes of Health Stroke Scale; mRS: modified Rankin Scale; cCoW: complete Circle of Willis; vCoW: Variants of the Circle of Willis.

a Mann-Whitney U-test for continuous variables and chi-squared test or Fishers exact test for categorical variables.

b Both history of atrial fibrillation and newly diagnosed atrial fibrillation on admission or during hospital stay.

### Affected arterial territories

Altogether, 386 patients had lesions in the right anterior territory, 424 had lesions in the left anterior territory, and 382 patients had lesions in the posterior territory. Of patients with lesions in the posterior territory, 84 had isolated lesions in the PCA territory.

### Stroke lesion patterns

More-than-one territory stroke pattern was seen in 238 (24.0%) patients. In patients with fPCA, more-than-one territory stroke patterns according to Tatu maps were less common than in patients with adult PCA (61/330 (18.5%) vs. 172/640 (26.9%), p = 0.014). In patients with anterior variants, frequency of more-than-one territory stroke pattern was similar to patients without anterior cCoW (19/67 (28.4%) vs. 219/924 (23.7%), p = 0.459).

### Reclassifying stroke patterns

Of 238 patients initially categorized as having more-than-one territory stroke pattern, 20 (8.4%) were reclassified into having one territory lesion pattern after correcting for vCoW. All of these patients had fPCA. Stroke lesions originally presumed to belong to the vertebrobasilar territory had to be re-assigned to the ICA territory. More-than-one arterial territory stroke lesion had only been mimicked since all lesions were distal to a common (ICA) artery (Figure 1). Of the 20 patients affected by the reclassification from more-than-one to one territory pattern, six (30%) had a severe symptomatic carotid stenosis. Out of 84 patients with isolated lesions in the PCA territory, 11 had fPCA (13.1%). These cases were reclassified from posterior to anterior arterial territory. No clinically relevant ipsilateral ICA stenosis was found in any of these patients.

Reclassification from originally presumed one territory to more-than-one territory stroke pattern was indicated in 4/753 (0.5%) patients. All these patients showed vCoW in the posterior circulation (Figure 2).

## Discussion

In this cohort of patients with acute ischemic stroke, MRA revealed at least one vCoW in 37.1% patients with most variants observed in the posterior circulation (33.3% compared to 6.7% in the anterior circulation). The adjustment of large cerebral vessel territories after considering vCoW led to several reclassifications of lesions patterns with relevance for the assumed ischemic stroke etiology.

Reports on the frequency of vCoW are available from autoptic examinations and neuroimaging studies using CTA or MRA.^5,8–11^ Depending on the method, the criteria used for defining variants and the examined population previous studies reported frequencies of any vCoW ranging from 45% to 88%.^8–15^ Our findings on the frequency of fPCA are in accordance with most previous studies, which found fPCA in around 20–30% of cases.^9,15–19^ The higher frequency of fPCA on the right side found in our study was also reported by two earlier studies in populations with cerebrovascular diseases.^9,16^ In line with earlier studies, we found a lower frequency of vCoW in the anterior circulation compared to the posterior circulation.^9,13,15^ Similar to the prevalence of 6.7% of agenesis or hypoplasia of the A1 segment of the ACA in our study, other imaging and autopsy studies reported the prevalence of this variant ranging from 9.5 to 11.7%.^6,9,15,20^

To our knowledge, this is the first study that examined rates of reclassification of stroke patterns in patients with vCoW. The detection of vCoW led to stroke pattern reclassification in 8.9% of patients. The majority were reclassified from more-than-one territory pattern to one territory stroke pattern. A minority of patients was reclassified from one to more-than-one-territory lesion pattern after taking vCoW into consideration. We showed that more-than-one territory stroke pattern is overdiagnosed in a relevant number of patients when vCoW is not taken into account. Consequently, proximal embolic sources may be preferably searched for as an underlying cause of the ischemic stroke. Although the combination of having stroke in the anterior (MCA + ACA) and PCA territories in the setting of a fPCA and symptomatic carotid stenosis was rare in our study (six out of nine hundred ninety-one cases), it is important to identify such patients, as they may benefit from carotid endarterectomy in cases of carotid artery disease. Current guidelines on the early management of patients with acute stroke recommend performing CTA and MRA in selected patients with suspected large vessel occlusion (i.e. patients with severe neurological symptoms, expressed by a higher NIHSS), who might benefit from mechanical thrombectomy.^21^ Hence, intracranial vessel imaging may not be performed in the acute phase in all patients, especially those with minor stroke symptoms. Without intracranial CTA or MRA in the acute phase, vCoW may be missed and stroke patterns may not be correctly classified. Etiological assessment of stroke lesions requires a comprehensive approach and should not be based solely on stroke patterns. We consider CTA or MRA of intra- and extracranial arteries as an indispensable part of diagnostic workup in all patients presenting with acute ischemic stroke. Failure to accurately determine the underlying cause of stroke may not only affect acute care but could also compromise choosing the optimal secondary prevention.

Some limitations must be taken into account, when interpreting our findings. Firstly, our study was retrospective, and the data were collected at a single site, potentially limiting generalizability of our findings. Secondly, only patients who were eligible for MRI were included in our study, possibly introducing a selection bias. Thirdly, the by and large single-person assessment may have introduced bias. Finally, our study did not control for intracranial vessel occlusions and stenoses, which could potentially influence the diameter of intracranial vessels and consequently change the anatomy of the CoW.

In conclusion, vCoW occur in more than a third of patients with ischemic stroke and are more common in the posterior than in the anterior circulation. Patients with vCoW in the posterior circulation are particularly prone to misclassifications of stroke patterns. We emphasize the importance of conducting intracranial vessel imaging in all stroke patients, independent of stroke severity, to facilitate correct classification of stroke etiology, and therefore provide optimal treatment.

## References

[1] Adams HP Jr Bendixen BH Kappelle LJ , et al. Classification of subtype of acute ischemic stroke. Definitions for use in a multicenter clinical trial. TOAST. Trial of Org 10172 in Acute Stroke Treatment. Stroke 1993; 24: 35–41. 7678184 10.1161/01.str.24.1.35

[2] Tatu L Vuillier F Moulin T . Chapter 13 Anatomy of the circulation of the brain and spinal cord. Handb Clin Neurol 2009; 92: 247–281. 18790279 10.1016/S0072-9752(08)01913-1

[3] Erdur H Milles LS Scheitz JF , et al. Clinical significance of acute and chronic ischaemic lesions in multiple cerebral vascular territories. Eur Radiol 2019; 29: 1338–1347. 30141060 10.1007/s00330-018-5684-8

[4] Hotter B Pittl S Ebinger M , et al. Prospective study on the mismatch concept in acute stroke patients within the first 24 h after symptom onset - 1000Plus study. BMC Neurol 2009; 9: 60. 19995432 10.1186/1471-2377-9-60 PMC3224745

[5] Gunnal SA Wabale RN Farooqui MS . Variations of anterior cerebral artery in human cadavers. Neurology Asia 2013; 18: 249–259.

[6] Ravikanth R Philip B . Magnetic resonance angiography determined variations in the circle of Willis: analysis of a large series from a single center. Ci Ji Yi Xue Za Zhi 2019; 31: 52–59. 30692833 10.4103/tcmj.tcmj_167_17 PMC6334567

[7] Padget DH. The circle of Willis. Its embryology and anatomy. In: Dandy WE, editor. *Intracranial Arterial Aneurysms*. New York, Comstock Publishing Company. 1944: 67–90.

[8] Kapoor K Singh B Dewan LI . Variations in the configuration of the circle of Willis. Anat Sci Int 2008; 83: 96–106. 18507619 10.1111/j.1447-073X.2007.00216.x

[9] Machasio RM Nyabanda R Mutala TM . Proportion of variant anatomy of the Circle of Willis and association with vascular anomalies on cerebral CT angiography. Radiol Res Pract 2019: 6380801. 31316832 10.1155/2019/6380801 PMC6601480

[10] Klimek-Piotrowska W Rybicka M Wojnarska A Wójtowicz A Koziej M Hołda MK . A multitude of variations in the configuration of the circle of Willis: an autopsy study. Anatom Sci Int 2016; 91: 325–333. 10.1007/s12565-015-0301-2 26439730

[11] Qiu C Zhang Y Xue C Jiang S Zhang W . MRA study on variation of the circle of willis in healthy Chinese male adults. Biomed Res Int 2015: 976340. 25629057 10.1155/2015/976340 PMC4299360

[12] Riggs HE Rupp C . Variation in form of circle of Willis. The relation of the variations to collateral circulation: anatomic analysis. Arch Neurol 1963; 8: 8–14. 13973856 10.1001/archneur.1963.00460010024002

[13] Krabbe-Hartkamp MJ van der Grond J de Leeuw FE , et al. Circle of Willis: morphologic variation on three-dimensional time-of-flight MR angiograms. Radiology 1998; 207: 103–11. 9530305 10.1148/radiology.207.1.9530305

[14] Hindenes LB Håberg AK Johnsen LH Mathiesen EB Robben D Vangberg TR . Variations in the Circle of Willis in a large population sample using 3D TOF angiography: the Tromsø Study. PLoS One 2020; 15: e0241373. 33141840 10.1371/journal.pone.0241373 PMC7608873

[15] Chen H-W Yen P-S Lee C-C , et al. Magnetic resonance angiographic evaluation of circle of Willis in general population: a morphologic study in 507 cases. Chin J Radiol-Taipei 2004; 29: 223–229.

[16] Shaban A Albright KC Boehme AK Martin-Schild S . Circle of Willis Variants: fetal PCA. Stroke Res Treat 2013: 105937. 23577277 10.1155/2013/105937 PMC3618940

[17] van Raamt AF Mali WP van Laar PJ van der Graaf Y . The fetal variant of the circle of Willis and its influence on the cerebral collateral circulation. Cerebrovasc Dis 2006; 22: 217–224. 16788293 10.1159/000094007

[18] Caldemeyer KS Carrico JB Mathews VP . The radiology and embryology of anomalous arteries of the head and neck. AJR Am J Roentgenol 1998; 170: 197–203. 9423632 10.2214/ajr.170.1.9423632

[19] Saeki N Rhoton AL Jr . Microsurgical anatomy of the upper basilar artery and the posterior circle of Willis. J Neurosurg 1977; 46: 563–578. 845644 10.3171/jns.1977.46.5.0563

[20] Perlmutter D and Rhoton AL Jr. Microsurgical anatomy of the anterior cerebral-anterior communicating-recurrent artery complex. *J Neurosurg.* 11976; 45: 259–272. . 10.3171/jns.1976.45.3.0259 948013

[21] Powers WJ Rabinstein AA Ackerson T , et al. 2018 Guidelines for the early management of patients with acute ischemic stroke: a guideline for Healthcare Professionals From the American Heart Association/American Stroke Association. Stroke 2018; 49: e46–e110. 29367334 10.1161/STR.0000000000000158

